# Reducing Hallucinations and Trade-Offs in Responses in Generative AI Chatbots for Cancer Information: Development and Evaluation Study

**DOI:** 10.2196/70176

**Published:** 2025-09-11

**Authors:** Sota Nishisako, Takahiro Higashi, Fumihiko Wakao

**Affiliations:** 1 Institute for Cancer Control, National Cancer Center, 5-1-1 Tsukiji, Chuo-ku, Tokyo, 104-0045, Japan, 81 3 3547 5201, 81 3 3547 8577; 2 Department of Public Health and Health Policy, Graduate School of Medicine, The University of Tokyo, Tokyo, Japan; 3 Headquarter for Cancer Information Services, National Cancer CenterTokyo, Japan

**Keywords:** artificial intelligence, AI, generative AI chatbot, generative pretrained transformer, GPT, retrieval-augmented generation, RAG, hallucination, medical information provision, cancer information service

## Abstract

**Background:**

Generative artificial intelligence (AI) is increasingly used to find information. Providing accurate information is essential to support patients with cancer and their families; however, information returned by generative AIs is sometimes wrong. Returning wrong information is called hallucination. Retrieval-augmented generation (RAG), which supplements large language model (LLM) outputs with relevant external sources, has the potential to reduce hallucinations. Although RAG has been proposed as a promising technique, its real-world performance in public health communication remains underexplored.

**Objective:**

This study aimed to examine cancer information returned by generative AIs with RAG using cancer-specific information sources and general internet searches to determine whether using RAG with reliable information sources reduces the hallucination rates of generative AI chatbots.

**Methods:**

We developed 6 types of chatbots by combining 3 patterns of reference information with 2 versions of LLMs. Thus, GPT-4 and GPT-3.5 chatbots that use cancer information service (CIS) information, Google information, and no reference information (conventional chatbots) were developed. A total of 62 cancer-related questions in Japanese were compiled from public sources. All responses were generated automatically and independently reviewed by 2 experienced clinicians. The reviewers assessed the presence of hallucinations, defined as medically harmful or misinformation. We compared hallucination rates across chatbot types and calculated odds ratios (OR) using generalized linear mixed-effects models. Subgroup analyses were also performed based on whether questions were covered by CIS content.

**Results:**

For the chatbots that used information from CIS, the hallucination rates were 0% for GPT-4 and 6% for GPT-3.5, whereas those for chatbots that used information from Google were 6% and 10% for GPT-4 and GPT-3.5, respectively. For questions on information that is not issued by CIS, the hallucination rates for Google-based chatbots were 19% for GPT-4 and 35% for GPT-3.5. The hallucination rates for conventional chatbots were approximately 40%. Using reference data from Google searches generated more hallucinations than using CIS data, with an OR of 9.4 (95% CI 1.2‐17.5, *P*<.01); the OR for the conventional chatbot was 16.1 (95% CI 3.7‐50.0, *P*<.001). While conventional chatbots always generated a response, the RAG-based chatbots sometimes declined to answer when information was lacking. The conventional chatbots responded to all questions, but the response rate decreased (36% to 81%) for RAG-based chatbots. For questions on information not covered by CIS, the CIS chatbots did not respond, while the Google chatbots generated responses in 52% of the cases for GPT-4 and 71% for GPT-3.5.

**Conclusions:**

Using RAG with reliable information sources significantly reduces the hallucination rate of generative AI chatbots and increases the ability to admit lack of information, making them more suitable for general use, where users need to be provided with accurate information.

## Introduction

Misinformation on the internet may cause harm to people with cancer, and this needs to be addressed. Currently, most patients with cancer face difficulties in obtaining accurate and reliable information from websites [1-3]. In Japan, many websites and social media platforms contain misinformation, and search engines display results based on access frequency, not accuracy [2,3]. Most patients with cancer do not verify the accuracy of the information owing to their health-related anxious psychological state [4]. Patients who receive complementary and alternative medicine with insufficient evidence have been reported to have poorer prognoses compared with those who receive standard cancer treatment, highlighting the potential impact that access to accurate information can have on cancer outcomes [5]. Now that many patients obtain information about their health from the internet [6-8], there is a pressing need to create a system that enables easy access to accurate medical information. Advances in artificial intelligence (AI) have been applied to medicine to assist in diagnosis [9], particularly in interpreting medical images in areas such as radiology, pathology, gastroenterology, and ophthalmology [10-16]. In information retrieval, generative AI chatbots using natural language processing have been reported to have advantages over traditional search engines [17-21]. Using generative AI chatbots in searching for cancer-related information has the potential to provide patients with easily accessible and understandable health information.

A major issue with generative AI responses is their potential to provide incorrect yet seemingly plausible information, known as hallucination [22,23]. To reduce the rate of hallucinations, a method called retrieval-augmented generation (RAG) has been developed [22,24,25]. RAG combines nonparametric memory (external knowledge bases), specialized databases, and internet information with the training data of large language models (LLMs) [24-26]. This approach provides up-to-date knowledge and generates responses based on relevant evidence, minimizing hallucinations [26]. Therefore, we hypothesized that an AI chatbot with RAG that uses text from the cancer information service (CIS) [27] could provide accurate cancer-related information to the general public and patients. CIS is a reliable cancer information website operated by the National Cancer Center of Japan. The content is developed by medical professionals, including physicians, and is thoroughly reviewed before publication.

This study aims to examine how generative AI chatbots that refer to reliable information respond and explore the potential of AI in providing trustworthy cancer information. We developed a prototype of a generative AI chatbot that uses specific reference information and compared it to a traditional generative AI chatbot that relies only on its pretraining data, without any references. We investigated how RAG can help reduce these hallucinations in LLMs when delivering cancer-related medical information by assessing the characteristics of their responses and hallucination rates.

## Methods

### Ethical Considerations

This research does not involve human participants who require protection from risks involved in research. Therefore, it does not need a review by an institutional review board, according to the Japanese ethics guidelines for medical research involving human participants [28]. This study was reported in accordance with the TRIPOD (Transparent Reporting of a Multivariable Prediction Model for Individual Prognosis or Diagnosis)-LLM [29].

### Development of Generative AI Chatbots

**Figure 1.**
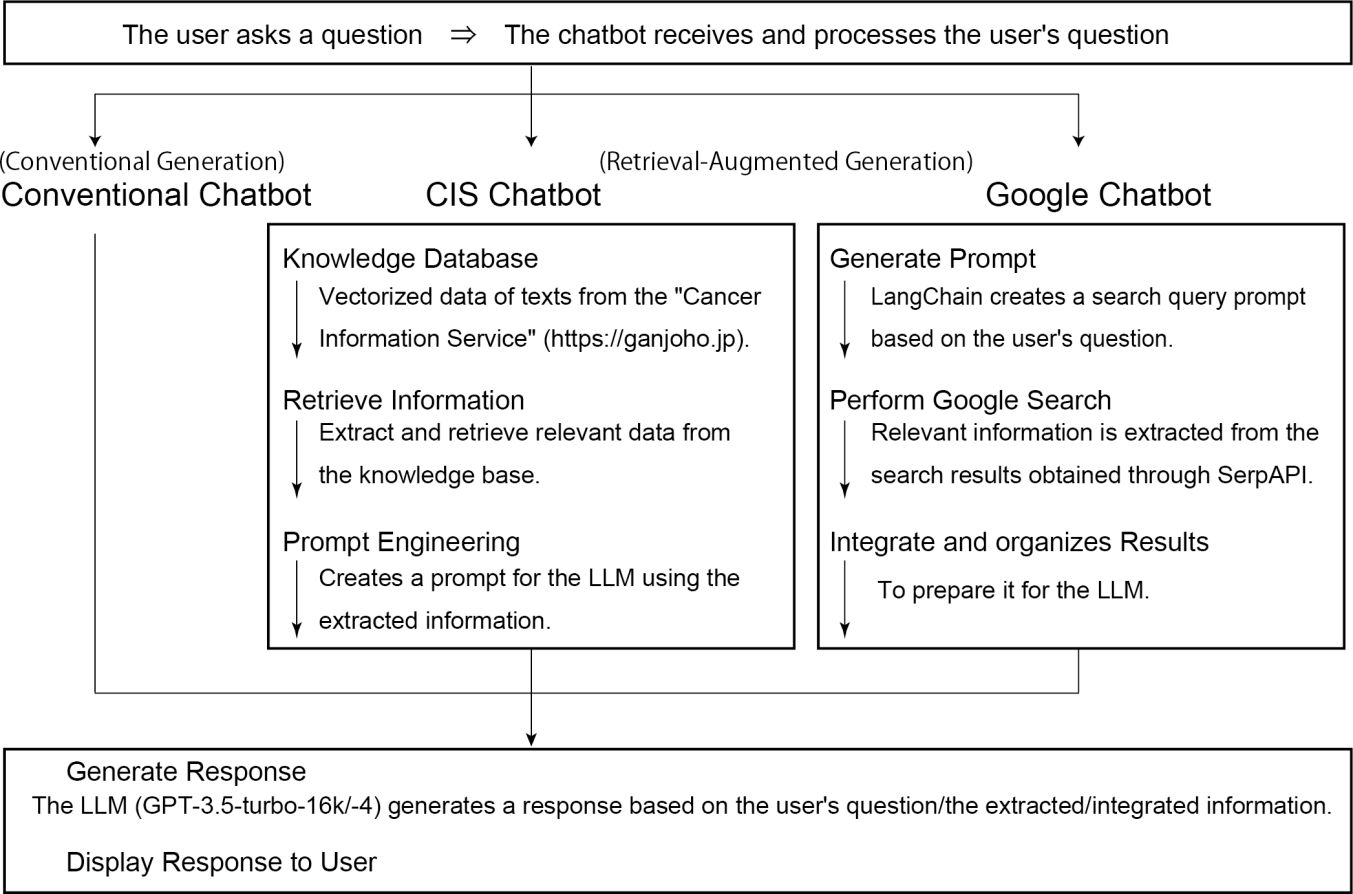
Overview of 3 artificial intelligence (AI) chatbots. Six types of AI chatbots using retrieval-augmented generation (RAG). In the CIS chatbot system, reference information sources include “Cancer Information Service”, a reliable cancer information website operated by the National Cancer Center. In the Google chatbot system, responses are generated based solely on information retrieved from Google search results. The conventional chatbot uses the generative pretrained transformer without RAG. All generative AI chatbots used GPT-4 and GPT-3.5-turbo-16k. CIS: cancer information service; GPT: generative pretrained transformer; LLM: large language model.

As useful methods to reduce hallucinations, RAG, fine-tuning, and prompt engineering have been reported [25]. This study adopted RAG, which has the advantage of shorter development time, being simpler and more cost-effective than fine-tuning, and being easier to develop [25]. Additionally, the concept of the developed RAG is considered to be more easily applicable to other areas compared with prompt engineering [24,25]. We developed 6 types of generative AI chatbots by combining 2 versions of LLMs (GPT-4 and GPT-3.5 turbo-16k, referred to as GPT-3.5) with 3 reference information sources using RAG (CIS, Google search, and none) (Figure 1). Accordingly, the resulting chatbot types were as follows: CIS-based chatbots using GPT-4 and GPT-3.5, Google-based chatbots using GPT-4 and GPT-3.5, and conventional chatbots without RAG using only LLM, GPT-4, and GPT-3.5. The CIS chatbot system used CIS as the reference information source. We selected CIS as the RAG information source because its content is highly accurate and user evaluations indicate a sufficiently high overall satisfaction, even when compared with global standards [30]. All web page data from CIS was converted to text using Python for Windows (version 3.12; Python Software Foundation) and processed using the BeautifulSoup library (version 4.12.3), on a per-page basis. The data was then vectorized using text-embedding-ada-002 (OpenAI, Inc, accessed between March 15 and 20, 2024) and stored in a knowledge database (Azure AI Search, Microsoft Corporation, Redmond, WA, USA). To retrieve similar texts in response to user queries, a vector search was conducted using the Best Matching 25 algorithm and the Hierarchical Navigable Small World algorithm. The retrieved text was then processed by passing it to the LLM via RAG with prompt engineering, using an application programming interface (API) (OpenAI, Inc.) to generate responses. The Google chatbots were developed to compare responses generated by chatbots using accurate information sources (CIS) with those generated based on results from traditional search engines. These chatbots generated responses based solely on information retrieved from Google search results. The system used SerpAPI (integrated via LangChain, New York, NY, USA) to execute search queries and obtain relevant search results, including top-ten ranking websites. The conventional chatbots used GPT without any specific references, generating responses through the API. The system environment was developed using Python and Google Colaboratory (Google LLC, Mountain View, CA, USA), with the project development commissioned to Pipon Inc. (Tokyo, Japan).

### Datasets

The questions were designed with the assumption that they would be used by the general public; thus, they did not cover specialized medical knowledge. Questions related to clinical matters and daily life were selected. One of the authors (SN) collected 80 cancer-related questions from 2 sources—the CIS website and Yahoo! Answers [31], a general opinion-based, community-driven platform provided by Yahoo Japan Corporation that is used to obtain information and resolve inquiries—between May and June 2024. All collected questions were reviewed by the authors. The questions were selected based on the following criteria: information availability, practical relevance to daily life, and simplicity of structure. These questions were generated by converting CIS and Yahoo! Answers content into interrogative forms, such as “What is…?” or “Could you explain…?.” After removing duplicates and integrating similar items, the final set comprised 62 cancer-related questions written in Japanese (Multimedia Appendix 1). Of these, 31 questions were based on information available on the CIS website. The remaining 31 questions addressed topics not covered by the CIS website. These were created by referencing posts on Yahoo! Answers. Questions were formulated by analyzing multiple user-submitted entries to capture common concerns and frequently asked issues among the general public. For each question from Yahoo! Answers, the absence of corresponding information on the CIS website was confirmed before inclusion. Each question was entered as a prompt, including the original full text in Japanese, along with a standardized instruction: "Use the following pieces of context to answer the question. If you don’t know the answer, just say that you don’t know; don’t try to make up an answer.” Only one response was generated per question without regeneration or manual revision. AI chatbot responses to these questions were generated by entering prompts (including the full original text of the questions). All 62 questions were fed into the 6 chatbots, yielding a dataset of 372 entries, and results were recorded in a data spreadsheet and linked to the corresponding questions. Access to all AI chatbots was provided between May and September 2024. For the English version presented in this paper, the responses in the dataset were translated from Japanese using GPT-4o and manually checked for technical terms by the authors. The original Japanese texts are shown in Multimedia Appendix 1.

### Data Analysis

All generated responses were reviewed for accuracy and absence of hallucinations by one of the authors with 8 years of work experience at a university hospital, with practical involvement in patient support and medical communication that includes patients with cancer. Any ambiguity was resolved by consulting another author, a physician with more than 20 years of cancer information experience with the National Cancer Center of Japan. Chatbot responses were primarily classified into 5 types (Table 1): “No response,” “No response but provided additional information,” “Unclear response,” “Response containing hallucinations,” and “No issues.” Under no response, the chatbot responded, “Unable to respond due to text not containing information,” which was categorized as “No response.” In contrast, the case where some additional information was provided as “Unable to respond, please consult your doctor or cancer consultation and support center” was categorized as “No response but provided additional information.” Some of the responses provided were observed as unclear, including phrases like “It is not clear...” or “It is said to...,” which were categorized as “Unclear responses,” while others presented unsupported statements, including names that do not exist. Responses that included fictional names or terms, as well as incorrect information—such as nonevidenced answers or errors regarding surgical indications—were categorized as “Responses containing hallucinations.” We defined hallucinations as responses that included nonexistent events, names, or terms, and unsubstantiated answers, distinguishing them from unclear responses. It is important to provide accurate information in the medical field; therefore, we defined a hallucination as a response that risks causing medical harm. Even when a response did not explicitly make a statement, if it could potentially cause medical harm, for example, “Consuming a lot of sugar may potentially increase the growth of cancer…,” it was classified as a hallucination. Conversely, if the response was unlikely to have significant effects, such as “Exercising while having cancer can be effective if done under proper guidance and the supervision of a physician…,“ it was classified as an “Unclear response.” All other responses were classified as “No issues.”

**Table 1.** Examples of responses to chatbot queries and classification.

Questions and responses^a^	Classification
Q: "I live in the Kita Ward of Sapporo City. A friend has been diagnosed with bile duct cancer. Which hospital would be recommended for treatment?”	
A: “The provided text does not contain information about hospitals in Kita Ward of Sapporo City.” (Generated by Google Chatbot GPT-4, CIS^b^ Chatbot GPT-4)	No response
A: "The provided text does not contain information about hospitals in Kita Ward of Sapporo City. However, contacting the ’Cancer Consultation Support Center’ may be helpful, as their staff can assist in finding hospitals with experience in treating bile duct cancer.” (CIS Chatbot GPT-3.5)	No response but provided additional information
A: “There are hospitals and clinics in Sapporo City, Hokkaido, that specialize in the diagnosis and treatment of bile duct cancer.” (Google Chatbot GPT-3.5)	Unclear response
A: “Here are some hospitals in the Sapporo City Kita Ward area that are known to provide treatment for bile duct cancer: Hokkaido University Hospital Sapporo Medical University Hospital Sapporo City Hospital” (nonexistent name) (Conventional Chatbot GPT-4,‐3.5)	Response containing hallucinations
Q: "Can garlic treat cancer?”	
A: “While it is not clear whether garlic is effective in the treatment of cancer, it is said to have potential cancer-preventive effects.” (Google Chatbot GPT-3.5)	Unclear response
Q: " Could you tell me which mushroom is most effective against cancer?”	
A: “The mushroom known to be effective against cancer is the Turkey Tail mushroom.” (Nonevidence-based information) (Conventional Chatbot GPT-4, Google Chatbot GPT-4,‐3.5)	Response containing hallucinations

a The response was given in Japanese and presented in English in the table.

b CIS: cancer information service.

The response rate for model output and the hallucination rates of different chatbots were compared using a Fisher exact test, and the subgroup analysis was conducted with questions on and not on the CIS website. To assess the effect of RAG in reducing hallucinations, a generalized linear mixed-effects model (GLMM) assuming a binomial distribution on all datasets was applied. In this model, the reference data (ie, CIS vs conventional/Google) and versions of LLMs (ie, GPT-4 vs GPT-3.5) were included as fixed effects. The model was adjusted for whether the question content was covered on the CIS website, and both the presence of CIS-covered questions and response length were included as random effects. In the process of constructing the model, it was confirmed via Akaike information criterion comparison that these 2 random-effect factors did not significantly influence the model outcome. Odds ratios (OR) for hallucinations associated with the explanatory variables included as fixed effects were estimated using GLMM. Sample size calculations were based on a pilot dataset comprising 1000 simulation runs. The effect size assumed was an OR of 9.54, which was derived from the pilot testing with the preliminary samples of 240. The statistical power was set to β=.8, and α=.05. The calculation showed that we needed a sample size of 372. Although the observed OR was 9.4, a little smaller than the assumed effect size, we had a power of β of 85.6% (95% CI 83.3‐87.7). In this analysis, the collected categorical data were transformed into dummy variables, and continuous variables were dichotomized with the median for use as categorical data. The normality of the data was assessed using the Shapiro-Wilk test and a normal Q-Q plot. Statistical analyses were conducted using Stata/IC for Windows (version 16.0; StataCorp LP). We used R for Windows (version 4.2.1; R Foundation for Statistical Computing) for the sample size calculation in GLMM (*simr* package) and modeling (*lme4* package). All reported *P* values were 2-sided, and *P*<.05 was considered statistically significant.

## Results

### Chatbot Response Rates and Patterns

**Figure 2.**
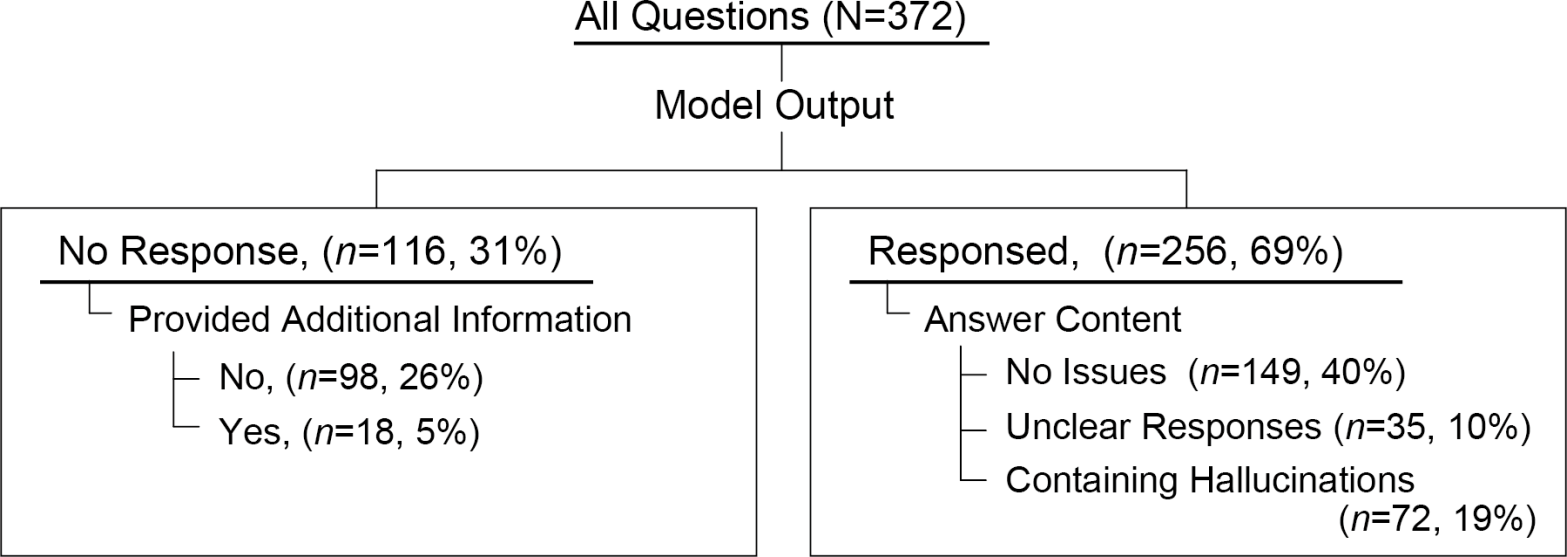
Categorization of AI chatbot responses to all questions. Chatbot responses were categorized as follows: “No response” for cases where it stated, “Unable to respond due to text not containing information,” and “No response but provided additional information” for responses like, “Unable to respond, please consult your doctor or cancer consultation and support center.” “Unclear responses” included phrases like, “It is not clear." or “it is said to.," while “Responses containing hallucinations” referred to fictional names, unsupported claims, or incorrect information, such as errors about surgical indications. All other responses were classified as “No issues.”

Responses to 372 questions were classified according to the definitions described in the methods section. Of these responses, 116 (31%) were categorized as “No response,” with 18 (5%) being “No response but provided additional information.” Of the remaining 256 (69%) responses, 35 (10%) were “Unclear response,” and 72 (19%) were “Responses containing hallucinations.” Furthermore, 149 (40%) of all responses did not contain any issues and were classified as “No issues” (Figure 2).

Table 2 shows the proportions of responses/nonresponses to the questions. The response rates varied significantly across the 6 chatbots, ranging from 22 (36%) to 62 (100%) responses out of 62 questions. The chatbots with RAG (ie, CIS chatbot and Google chatbot) had lower response rates than the conventional models. For 31 questions on information on the CIS website, the responses of the CIS chatbots were 22 (71%) for GPT-4 and 30 (97%) for GPT-3.5; however, they correctly returned nonresponse for questions not covered by CIS (0% response). On the other hand, Google chatbots responded to both questions on information covered and not covered by CIS more closely—14 (45%) and 16 (52%) for GPT-4, and 28 (90%) and 22 (71%) for GPT-3.5. The conventional chatbot responded to all questions regardless of the LLM versions.

**Table 2.** Response rates to questions by generative artificial intelligence (AI) chatbots for information covered/not covered by the cancer information service (CIS).

AI chatbot /LLM^a^^b^	Total (N=62), answered, n (%)	Covered by the CIS (n=31), answered, n (%)	Not covered by the CIS (n=31), answered, n (%)	*P* value^c^
CIS GPT-4	22 (36)	22 (71)	0 (0)	<.001
CIS GPT-3.5	30 (48)	30 (97)	0 (0)	<.001
Google GPT-4	30 (48)	14 (45)	16 (52)	.80
Google GPT-3.5	50 (81)	28 (90)	22 (71)	.11
Conv. GPT-4	62 (100)	31 (100)	31 (100)	>.99
Conv. GPT-3.5	62 (100)	31 (100)	31 (100)	>.99

a The data referenced by the tested chatbot is as follows: CIS refers to a custom database with the National Cancer Center’s “Cancer Information Service;” Google refers to websites ranked highly by the Google search engine; Conventional GPT is based on conventional training data with no specific reference. GPT-3.5 refers to GPT-3.5-turbo-16K.

b LLM: large language model.

c Analyzed with Fisher exact test.

### Chatbot Hallucinations

Table 3 shows the proportions of responses with hallucinations for the six chatbots. AI chatbots with RAG showed fewer hallucinations than conventional chatbots. Chatbots that reference CIS generated the lowest rate of hallucinations. For 31 questions on information on the CIS website, hallucinations seldom occurred: 0 (0%) and 2 (6%) for CIS chatbots and 2 (6%) and 3 (10%) for Google chatbots with GPT-4 and 3.5, respectively. For 31 questions on information not covered by CIS, Google chatbots generated more responses containing hallucinations: 6 (19%) with GPT-4 and 11 (35%) with GPT-3.5. The conventional models generated hallucinations in approximately 40% of their responses. Significant differences in the proportion of responses containing hallucinations were observed between the six types of chatbots (all *Ps*<.001).

**Table 3.** Rate of responses containing hallucinations (defined as text containing fictitious names, nonexistent terms, or incorrect information) by generative artificial intelligence (AI) chatbots for questions about information covered/not covered by the Cancer Information Service (CIS).

AI Chatbot /LLM^a^^b^	Total (N=62), occurrence, n (%)^c^	Covered by the CIS (n=31), occurrence, n (%)^c^	Not covered by the CIS (n=31), occurrence, n (%)^c^
CIS GPT-4	0 (0)	0 (0)	N/A^d^
CIS GPT-3.5	2 (3)	2 (6)	N/A
Google GPT-4	8 (13)	2 (6)	6 (19)
Google GPT-3.5	14 (23)	3 (10)	11 (35)
Conv. GPT-4	23 (37)	12 (39)	11 (35)
Conv. GPT-3.5	25 (40)	15 (48)	10 (32)

a LLM: large language model.

b The data referenced by the tested chatbot is as follows: CIS refers to a custom database with the National Cancer Center’s “Cancer Information Service;” Google refers to websites ranked highly by the Google search engine; Conventional GPT is based on conventional training data with no specific reference. GPT-3.5 refers to GPT-3.5-turbo-16K.

c *P*<.001 (analyzed with Fisher exact test).

d Not applicable due to no response.

### Factors Associated With the Generation of Hallucinations

In bivariate analysis based on 256 questions that received responses (Figure 2), hallucinations differed significantly in their rate of occurrence depending on the RAG reference data (Table 4): the lowest rate was observed in the CIS chatbot, with hallucinations occurring in 2 (2%), followed by the Google chatbot with 22 (18%) and conventional chatbot with 48 (39%) (*P*<.001). The occurrence of hallucinations was not related to the content of questions being covered or not covered by CIS, output length, and versions of LLM (All, *P*≥.05). In multivariate analysis, adjusting for other factors, models with RAG reference data from Google search results were more likely to generate hallucinations compared with those with RAG reference data from CIS, with an OR of 9.4 (95% CI 1.2‐17.5; *P*<.01). Similarly, conventional GPTs had an OR of 16.1 (95% CI 3.7‐50.0; *P*<.001) compared to those with RAG reference data from CIS. Differences in LLM versions did not contribute to hallucination generation.

**Table 4.** Factors associated with the generation of responses containing hallucinations.

Prognostic factor^a^	Bivariate models^b^		Multivariate models^c^	
	n (%)	*P* value	OR^d^ (95% CI)	*P* value
Questions that received responses (n=256)	72 (19)	—^e^	—	—
Content of questions		.60	—	—
Covered by the CIS^f^	34 (18)			
Not covered by the CIS	38 (20)			
The length in the output^g^ (words, n)		.05	—	—
5‐216	29 (23)			
217‐784	43 (34)			
Versions of LLM^h^^i^		.19		.54
GPT4	31 (17)		1 (reference)	
GPT3.5	41 (22)		1.2 (0.6‐2.0)	
Reference data^j^		<.001		
CIS	2 (2)		1 (reference)	—
Google	22 (18)		9.4 (1.2‐17.5)	<.01
Conv. GPT	48 (39)		16.1 (3.7‐50.0)	<.001

a Mean variance inflation factor is 1.2 (range 1.0‐2.4).

b Analyzed with the chi-square test.

c Analyzed with a generalized linear mixed-effect model adjusted for the type of LLM and reference data (fixed effect), content of question and the length in the output (random effect).

d OR: odds ratio.

e Not applicable.

f CIS: cancer information service.

g Count of characters in Japanese.

h GPT-3.5 refers to GPT-3.5-turbo-16K.

i LLM: large language model.

j The data referenced by the tested chatbot is as follows: CIS refers to a custom database with the National Cancer Center’s “Cancer Information Service” ; Google refers to websites ranked highly by the Google search engine; Conventional GPT is based on conventional training data with no specific reference.

## Discussion

### Principal Findings

We focused on reducing misinformation by avoiding hallucinations. By testing generative AI chatbots with RAG on different referencing datasets, our study showed that incorporating reliable cancer information significantly reduced the occurrence of hallucinations compared with using conventional GPT models or Google searches as reference sources. Conversely, when no reference information was available, the system correctly returned no response.

### Comparison to Prior Work

In this study, nonparametric memory LLMs (CIS Chatbot/Google chatbot) had fewer hallucinations than the parametric memory LLM (conventional chatbot) (2%‐18% vs39%, *P*<.001, Table 4). This highlights the importance of the quality of nonparametric knowledge sources, as indicated by the Google chatbot with the odds of hallucination 9.4 (95% CI 1.2‐17.5, *P*<.01) times as large as that of the CIS chatbot (Table 4). Many websites that provide information on cancer treatment have a higher proportion of harmful information compared with those offering reliable information in Japanese [2,3]. In this study, the responses by the Google chatbot to information that lacks evidence (not covered by CIS) and its higher incidence of hallucinations compared with those of the CIS chatbot suggest that misinformation or evidence-lacking information existed within the reference sources. Since LLMs generate responses based on reference information without assessing the accuracy of that information, the accuracy of the RAG information source is crucial for providing accurate information. The use of AI chatbots on various cancers has been reported, including head and neck cancer [32], prostate cancer [33], liver cancer [34], kidney cancer [35], breast cancer [36,37], lung cancer [20], colon cancer, pancreatic cancer [38], cervical cancer [39], as well as topics like cancer treatment [13,40], ophthalmic information [11,41,42], and cardiovascular disease prevention [43]. In these studies, RAG was not used. In situations where the definitions and extent of hallucinations are not clearly established, AI chatbots generally lack expertise and accuracy, despite containing hallucinations and significant gaps in information [15,44]. Clinical acceptability can be influenced by many factors, including social expectations of the AI, the extent of damage the potential inaccuracy can cause, and the support system to check and correct hallucinations. Additionally, previous studies in which national medical licensing examination questions were answered by a chatbot have reported that the accuracy of AI responses varies depending on the complexity of the questions [45]. The potential influence of clinical informatics and cancer-related topics, which could be more prone to hallucinations compared with other clinical, medical, and health informatics topics, could not be thoroughly examined in this study. The RAG-AI chatbot could enable the bridging of these gaps if it obtains accurate information. Overall, our findings support that in RAG-based systems, the reliability of reference information plays a key role in reducing hallucination generation.

Generally, questions from patients with cancer span a wide range of topics and are not limited to evidence-based content [46]. In this study, a trade-off was observed between the hallucination rate and response rate across different types of chatbots. The conventional chatbot responded to all inquiries, the RAG chatbot, especially with CIS reference, exhibited an increased rate of unanswered questions (100% vs 36‐81%, *P*<.001, Table 2). Not providing misinformation suggests that developing RAG-AI chatbots has the potential to prevent patients from being harmed by misinformation. We need to consider how such systems should address responses that lack scientific evidence during their development for general use. Conversely, we observed that the CIS chatbot at times did not respond to questions about information that existed in the CIS references. Cases in which the chatbot does not respond despite the presence of relevant information in the reference data represent a technical challenge that must be addressed. These problems may have been caused by errors in data retrieval by the LLM or by vectorization or structuring in the knowledge database [22]. However, these could be minimized through LLM fine-tuning and prompt engineering [22]. When developing RAG-AI chatbots, information from reliable data sources can be used to explain the reasons for lack of evidence or to issue warnings by combining a scenario-based chatbot with standardized responses [47]. Further, the AI chatbot search style used in this study could display only relevant content according to the user query and has the added benefit of simplifying the information retrieval process, improving efficiency, and saving time [15].

### Limitations

This study has several limitations. First, the evaluated LLMs were limited to GPT-3.5-turbo-16k and GPT-4. AI advances are rapid, and as of 2025, many new capable versions, such as GPT-4.5 [48], Gemini 2.0 Pro [49], and Claude 3.7 Sonnet [50] have been developed. While there were no statistically significant differences in hallucination rates between the LLM versions, each LLM has its own characteristics, and there may be models better suited for RAG-AI chatbots. Second, we did not verify the accuracy of the data referenced by the RAG of the Google chatbot. Because the Google chatbot references the Google search results, the information referenced by the Google chatbot naturally included treatments not approved by the Japanese regulatory authorities and did not exclude sites that made exaggerated claims without relevant clinical evidence. A report showed that advertisements constitute 10% of the information accessed in search results in Japan [3]. In the future, analyzing the relationship between the original reference data and the generated responses could provide insights into the hallucination generation process. Third, all questions and responses were conducted in Japanese. Reports indicate that GPT-4 has improved capabilities for languages other than English compared with GPT-3.5 [51]. However, since we did not compare responses in English, some hallucinations may have been caused by simple errors in translation into Japanese. Nonetheless, the results indicate that RAG, using accurate information sources, can generate high-quality responses even in languages other than English. Fourth, in this study, a wide range of CIs were observed in the multivariate model (eg, conventional GPT OR 16.1, 95% CI 3.7‐50.0). This may reflect that the sample size was relatively small. A model excluding the length of the generated text and quality of the questions showed that the Akaike information criterion remained almost unchanged compared with the final model. Investigating a larger sample size in the future may lead to more robust results. Finally, having a single reviewer for response classification may have introduced some misclassification bias. Of the 76 detected hallucinations, 51 clearly contained factually incorrect information, such as nonexistent events, names, or terms. These errors were objectively confirmed by cross-checking with the CIS website and medical guidelines. The remaining 25 cases included statements presented with certainty despite lacking medical evidence, raising concerns about their reliability. These cases were reviewed by a physician with extensive experience in cancer information to ensure the validity of the assessments. Accordingly, it is reasonable to assume that the risk of misjudgment associated with single-reviewer classification was minimal within the scope of this study.

### Future Directions

The development of RAG-AI chatbots such as the CIS chatbot in this study, which is linked to highly reliable information, like medical guidelines and scientific papers, has the potential to address these issues. Such systems have the potential to bridge the current gaps in accessing medical information for patients with cancer and their families.

### Conclusions

This study demonstrates that using RAG to incorporate accurate medical information into generative AI chatbots can reduce hallucinations. We believe that the results of this study are encouraging. Advances in both generative AI technology and better information sources can generate optimal responses for topics that lack scientific evidence while preventing hallucinations due to inferior data use, enabling accurate information provision by AI chatbots.

## Supplementary material

10.2196/70176 Multimedia Appendix 1 All Responses to Chatbot Queries.

## Abbreviations

AI: artificial intelligence
CIS: cancer information service
GLMM: generalized linear mixed-effects model
LLM: large language model
OR: odds ratio
RAG: retrieval-augmented generation
TRIPOD: Transparent Reporting of a Multivariable Prediction Model for Individual Prognosis or Diagnosis
